# Spin-configuration of emission states in zero-dimensional metal halides

**DOI:** 10.1093/nsr/nwae180

**Published:** 2024-05-25

**Authors:** Zhiyuan Kuang, Xinyu Huang, Xing Wang, Chengcheng Wang, Xinrui Wang, Wei Huang, Qiming Peng, Jianpu Wang

**Affiliations:** Key Laboratory of Flexible Electronics (KLOFE), Institute of Advanced Materials (IAM) & School of Flexible Electronics, Nanjing Tech University, Nanjing 211816, China; Key Laboratory of Flexible Electronics (KLOFE), Institute of Advanced Materials (IAM) & School of Flexible Electronics, Nanjing Tech University, Nanjing 211816, China; Key Laboratory of Flexible Electronics (KLOFE), Institute of Advanced Materials (IAM) & School of Flexible Electronics, Nanjing Tech University, Nanjing 211816, China; Key Laboratory of Flexible Electronics (KLOFE), Institute of Advanced Materials (IAM) & School of Flexible Electronics, Nanjing Tech University, Nanjing 211816, China; Key Laboratory of Flexible Electronics (KLOFE), Institute of Advanced Materials (IAM) & School of Flexible Electronics, Nanjing Tech University, Nanjing 211816, China; Key Laboratory of Flexible Electronics (KLOFE), Institute of Advanced Materials (IAM) & School of Flexible Electronics, Nanjing Tech University, Nanjing 211816, China; Strait Laboratory of Flexible Electronics (SLoFE), Fuzhou 350117, China; Key Laboratory of Flexible Electronics (KLOFE), Institute of Advanced Materials (IAM) & School of Flexible Electronics, Nanjing Tech University, Nanjing 211816, China; Key Laboratory of Flexible Electronics (KLOFE), Institute of Advanced Materials (IAM) & School of Flexible Electronics, Nanjing Tech University, Nanjing 211816, China; School of Materials Science and Engineering, School of Microelectronics and Control Engineering, Changzhou University, Changzhou 213164, China

**Keywords:** zero-dimensional metal-halides, spin-configuration, dual-peak emission, magneto-optical study

## Abstract

Understanding the spin-configuration of excited states in a luminescent material is essential for tailoring its properties for many applications such as light-emitting diodes and spin-optoelectronic devices. Zero-dimensional organic-inorganic metal halide (0D-OIMH) materials have demonstrated remarkable potential in diverse applications owing to their captivating optoelectronic characteristics. However, the electronic structure and spin-configuration of the frequently observed dual-peak emission in these materials remains a subject of intensive debate. In this study, we employ low-temperature magneto-optical measurements to investigate the excited state structure of a representative 0D-OIMH, namely (Bmpip)_2_SnBr_4_. The spin-configurations of the dark and bright states are clearly elucidated by measuring the magneto-polarization of the emissions. Our results reveal that the high-energy peak arises from bright excited states within a higher energy band, whilst the low-energy peak originates from a combination of triplet-bright states and singlet-dark states. These findings provide an unambiguous understanding of the exciton structures of the distinctive 0D-OIMHs.

## Teaser

Researchers discovered the spin-configuration of excited states in a promising zero-dimensional luminescent material, (Bmpip)2SnBr4, resolved a scientific debate with groundbreaking insights into its unique energy structures and shed light on designing better LEDs and spin-based devices.

## INTRODUCTION

Understanding the spin-configuration of excited states in a luminescent material is essential for tailoring its properties for many applications such as light-emitting diodes, spin-optoelectronic devices and quantum technologies with enhanced or novel functionalities. In organic semiconductors, where spin-orbital coupling (SOC) is almost negligible, the spin configuration of an exciton is solely determined by the spin directions of the electron and hole, leading to the formation of a bright singlet exciton and a dark triplet exciton [1]. Conversely, in many inorganic semiconductors, the orbital motion and spin of carriers are strongly coupled, breaking the conservation of spin. In such cases, the spin configuration of an exciton is influenced by the total angular momentum of the electron (*J*_e_) and hole (*J*_h_), resulting in a more complex determination. For instance, the lowest exciton in CdSe quantum dots exhibits a quintuplet spin-configuration, while excitons in CsPbI_3_ nanocrystals can manifest as either bright triplets or dark singlets [2,3].

Zero-dimensional organic-inorganic metal halide (0D-OIMH) materials, a kind of prospective luminescent materials with high photoluminescence quantum efficiency (PLQE), have shown great potential in optoelectronic applications and thus gathered significant attention recently [4–9]. Due to their intrinsic 0D electronic structure, these materials not only show a lot of advantages over 3D metal halides, such as a reduced sensitivity to defects and less self-absorption [10], but also own plentiful physical scenarios owing to their sensitivity to external fields such as mechanical force and heat [11,12]. 0D-OIMHs commonly show a dual-peak emission under photo-excitation, but the electronic structure and spin-configuration of the emission states remain a subject of intense debate. Although without evidence, previous studies commonly proposed that the low energy peak comes from the triplet dark state, while the high-energy peak comes from the singlet bright state, a lesson directly drawn from organic semiconductors [13,14]. Meanwhile, it is frequently claimed that the two peaks result from self-trapped excitons (STEs) and the corresponding free excitons [15].

In this study, we reveal the landscape of excited states in a typical 0D-OIMHs material, namely (Bmpip)_2_SnBr_4_ (with Bmpip representing 1-butyl-1-methylpiperidinium), via low-temperature magneto-optical measurements. The spin-configurations of the dark and bright states are clearly elucidated by measuring the magneto-polarization of the emissions. Our results demonstrate that the high-energy peak originates from bright excitons in a higher energy band, while the low-energy one is a convolution of triplet-bright and singlet-dark excitons. Our findings provide an unambiguous understanding of the exciton structures of the distinctive 0D-OIMHs.

## RESULTS AND DISCUSSION

**Figure 1.**
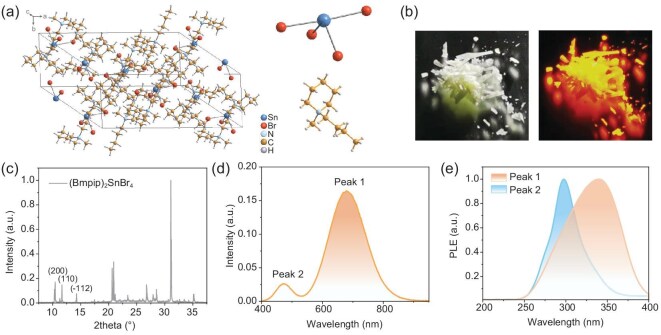
(a) Crystal structure of (Bmpip)_2_SnBr_4_. (b) Photographs of (Bmpip)_2_SnBr_4_ powder under illustrations of daylight and UV light (365 nm). (c) XRD spectrum of (Bmpip)_2_SnBr_4_ powder. (d) PL spectrum of (Bmpip)_2_SnBr_4_ excited by a 266 nm laser at room temperature. A low-energy Peak 1 and a high-energy Peak 2 can be observed. (e) PLE spectra of Peak 1 and Peak 2.

Figure 1(a) illustrates the crystal structure of (Bmpip)_2_SnBr_4_, showing that SnBr_4_^2−^ units disperse separately, with each unit surrounded by Bmpip^+^ cations. Figure 1(c) displays the XRD spectrum of (Bmpip)_2_SnBr_4_ powder, in which we can find typical diffraction signals from (200), (110) and (−112) crystal planes. Under 365 nm UV light illumination, the as-synthesized white powder shows a bright orange emission [Fig. 1(b)]. When excited by a 266 nm laser, a dual-peak emission is obtained, with a low-energy peak at 680 nm and a high-energy peak at 472 nm [Fig. 1(d)], consistent with previous reports [13]. Hereafter we label Peak 1 as the lower energy emission and Peak 2 as the higher energy one. The PL excitation (PLE) spectrum of Peak 1 differs from that of Peak 2 [Fig. 1(e)], implying that the two peaks have different origins.

**Figure 2.**
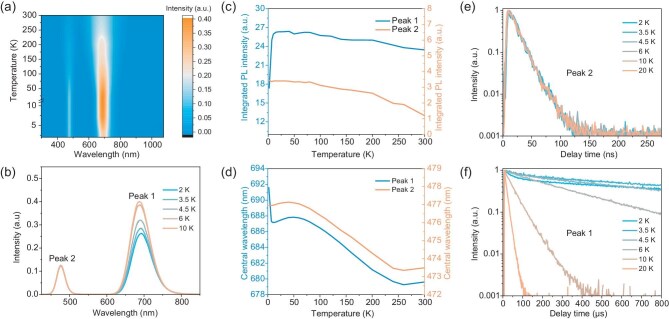
(a) Pseudo-color plot of the temperature-dependent PL spectra of (Bmpip)_2_SnBr_4_. (b) PL spectra of (Bmpip)_2_SnBr_4_ below 10 K. (c) Integrated PL intensities of Peak 1 and Peak 2 at different temperatures. (d) The central wavelengths of Peak 1 and Peak 2 as a function of temperature. (e and f) TRPLs of Peak 2 and Peak 1 at different temperatures.

The different origins of the two peaks can be further confirmed by temperature-dependent PL measurements. As shown in Fig. 2(a)–(c), the intensity of Peak 2 increases and tends to saturate as temperature decreases from 300 to 2 K. Above 10 K, the intensity of Peak 1 shows similar temperature-dependency as that of Peak 2, but it drops sharply as temperature further decreases to 2 K. Similarly, while the two peaks exhibit the same tendency with temperature in terms of wavelength shift above 10 K [Fig. 2(d)], Peak 1 undergoes a sudden red-shift as the temperature decreases below 10 K. These results clearly indicate different underlying mechanisms of the two peaks, which are further evidenced by transient PL (TRPL) measurements. As shown in Fig. 2(e) and (f), the TRPLs of Peak 2 remain unchanged at various temperatures, and the TRPL of Peak 1 exhibits a slow-down trend with temperature decreasing, along with the appearance of an additional ultra-slow decay component below 4.5 K. We note that these results are quite different from previous studies that were carried out in a narrower temperature regime from 77 K to room temperature [13,16], where Peak 1 was attributed to a pure dark state emission due to its monoexponentially long lifetime. The anomalous behavior of Peak 1 at ultra-low temperature suggests that more physical processes should be involved.

Traditionally, trap states emission [17,18] or low temperature-induced STE reduction [19,20] have been attributed to be the origin of the decreased PL intensity and increased PL lifetime at low temperatures. However, we can exclude these mechanisms as the cause of the anomalous behavior of Peak 1. Trap states emission will exhibit a saturation character under high excitation intensity. Whereas such a saturation tendency does not attend in the excitation intensity-dependent PL measurements [Fig. S2]. Low temperature-induced STE reduction, a typical mechanism due to the population reduction of phonons, will only slow down the TRPL, and cannot account for the sudden emerging ultra-slow decay component in the TRPLs below 4.5 K.

We propose that the anomalous behavior of Peak 1 arises from the occurrence of dark exciton emission at low temperatures, which can be verified by magneto-optical measurements. An external magnetic field would mix bright and dark states, increasing the oscillator strength of dark states and leading to the increased dark state emission with reduced lifetime [21–23]. Such measurement has also been introduced into low dimensional copper halide materials for distinguishing bright/dark excitons [24]. Therefore, the presence or absence of dark states can be discerned by monitoring the magneto-photoluminescence (MPL), which can be described as Equation 1 below [25–27]:


(1) \begin{eqnarray*}
{\mathrm{MPL}} = \frac{{I\left( B \right) - I\left( 0 \right)}}{{I\left( 0 \right)}}.
\end{eqnarray*}

Here *I*(*B*) and *I*(0) represent the PL intensities with and without an external magnetic field of *B*.

**Figure 3.**
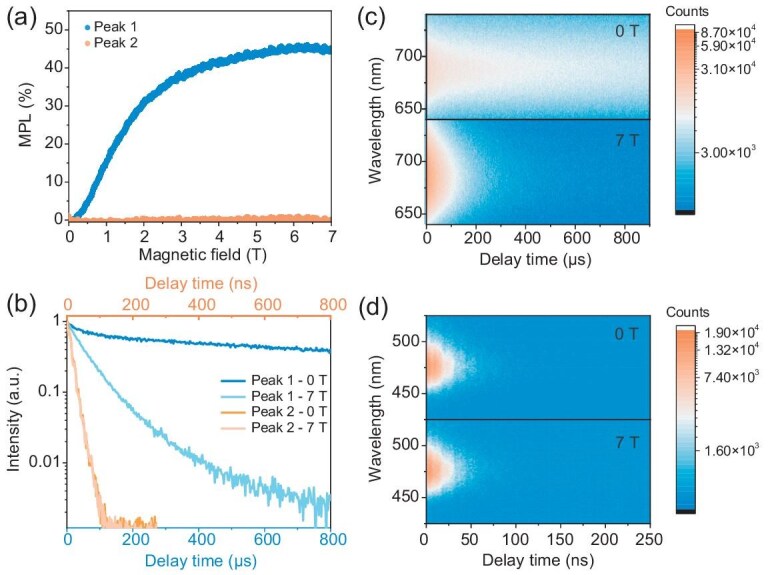
(a) MPLs of the two peaks at 2 K. (b) TRPLs of the two peaks with and without a magnetic field of 7 T at 2 K. (c) Pseudo-color plots of TRPLs of Peak 1 at 0 and 7 T. (d) Pseudo-color plots of TRPLs of Peak 2 at 0 and 7 T.

Figure 3(a) shows the MPLs of the two peaks at 2 K. The intensity of Peak 2 remains consistent under the external magnetic fields, indicating that Peak 2 is a purely bright state. While the intensity of Peak 1 exhibits significant MPLs of over 40% under high magnetic field. The MPLs of Peak 1 quickly decrease with the increase of temperature and diminish when temperature exceeds 6 K [Fig. S3]. We note that 6 K is also the transform temperature of the TRPL from double-exponential to mono-exponential [Fig. 2(f)]. The above results suggest that Peak 1 originates from bright states at high temperatures and is dominated by dark states at low temperatures. The nature of the two emission states can be further confirmed by magnetic field-dependent TRPL measurements at 2 K. As shown in Fig. 3(b), the lifetime of Peak 2 remains consistent with and without the magnetic field, whilst the lifetime of Peak 1 decreases greatly when the magnetic field is applied. The significantly shortened lifetime of Peak 1 is more clearly shown in the time-dependent PL spectra measurements in the presence and absence of a magnetic field of 7 T [Fig. 3(c) and (d)]. These results confirm the magnetic field-induced spin-mixing of bright and dark states, verifying that Peak 1 originates from a combination of spin-allowed bright state and spin-forbidden dark state.

**Figure 4.**
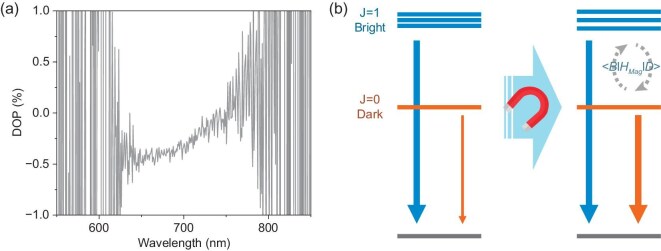
(a) DOP of (Bmpip)_2_SnBr_4_ at 2 K and 7 T. (b) The landscape and spin-configuration of emission states for Peak 1.

We further elucidate the spin-configuration of the dark and bright states by measuring the polarization of the emissions. At 2 K and 7 T, Peak 1 shows a clear difference between the right-handed (σ^+^) and left-handed (σ^−^) circularly polarized emissions, suggesting the existence of triplets [Fig. S4(c)]. The degree of polarization (DOP) is shown in Fig. 4(a), which is calculated from Equation 2 below [28,29]:


(2) \begin{eqnarray*}
{\mathrm{DOP}}\!\left( {\hbar \omega } \right) = \frac{{{{I}^ +\! }\left( {\hbar \omega } \right) - {{I}^ -\! }\left( {\hbar \omega } \right)}}{{{{I}^ +\! }\left( {\hbar \omega } \right) + {{I}^ -\! }\left( {\hbar \omega } \right)}}.
\end{eqnarray*}

Here *I*^+/−^(${\hbar \omega }$) refers to the intensity of σ^+/−^ emissions at a specific photon energy of ${\hbar \omega }$ (${\hbar }$ is the reduced Plank constant and *ω* is the angular frequency). We can find from Fig. 4(a) that under the external magnetic field of 7 T, the blue edge of Peak 1 shows a much higher DOP than that of the red edge (which is almost zero), indicating that Peak 1 comprises a bright triplet state with higher energy and a dark singlet state with lower energy. This assignment is confirmed by the unmeasurable DOPs in the absence of the magnetic field [Fig. S4]. The landscape and spin-configuration of emission states for Peak 1 is schematically depicted in Fig. 4b. Moreover, we believe that such spin-configuration of bright and dark states is due to the strong SOC induced by Sn [30–33] and are opposite to light-element–based organic light-emitting materials where the SOC can be neglected [34–36]. This can be supported by the calculated band structure and projected density of state (DOS) of the materials based on the first-principles theory, showcasing great differences in band-edge regions with and without SOC [Figs S5 and S6].

## CONCLUSION

In summary, we have unveiled the spin-configuration and electronic structure of excited states in a 0D-OIMH material ((Bmpip)_2_SnBr_4_) via low-temperature magneto-optical studies. Our findings clarify the ambiguity of the underlying mechanism of the commonly observed dual-peak emission in the 0D-OIMHs. Magnetic field effects on the PLs and TRPLs at low temperatures have demonstrated that the low-energy Peak 1 originates from both spin-triplet bright states and spin-singlet dark states, which contradicts previous assignment of Peak 1 as a pure dark state. For high-energy Peak 2, we suppose that it should result from the bright excitons in a higher energy band, not the lowest bright excited states or free excitons. These findings provide an in-depth understanding of the energy level structure of 0D-OIMH materials, thereby facilitating the advancement of these materials in the field of optoelectronics, such as white light illumination and ultraviolet light detectors.

## METHODS

**Material Synthesis.** To begin, 0.4 mmol BmpipBr and 0.2 mmol SnBr_2_ were dissolved in 1 ml EtOH. Then the precursor was heated to 80°C and kept for 12 h in order to obtain a clear solution. The resulting solution was cooled to room temperature slowly to gather crystals. The as-synthesized crystals were washed using EtO_2_ and then dried in a vacuum.

**PLE measurement.** PLE spectra were measured with Hitachi's fluorescence spectrophotometer (F-7100). For Peak 1 and Peak 2 we set the testing wavelengths as 680 and 472 nm, respectively.

**Temperature-dependent PL measurement.** The sample was placed in the chamber of an Oxford SpectromagPT for a low-temperature environment. A laser of 266 nm (EO-266-N, single pulse ∼100 μJ, pulse width ∼5 ns, from Changchun New Industries Optoelectronics Technology Co., Ltd.) was utilized for excitation and a UV-lens was used for light collection. Then we used a 400 nm-long pass filter to rule out the influence of the excitation laser and measured the PL spectra using an Ocean optics spectrometer (QE65).

**TRPL measurement.** The testing system is the same as the temperature-dependent PL measurement system. But the spectrometer here is substituted by the combination of spectrometer (ISOPlane160, Princeton Instruments) and EMICCD camera (PI-MAX4 512B).

**MPL measurement.** The magnetic field was supplied by the superconducting magnet system of Oxford SpectromagPT. Optical testing system is the same as temperature-dependent PL measurement. PL spectra were measured using Ocean optics spectrometer (QE65) while the magnetic field changes.

**Computational details.** Electronic band structures, density of states and structure relaxation calculations are performed with plane-wave pseudopotential approach implemented in DS-PAW package [37]. The exchange-correlation effects are recorded by PBE functional. The plane-wave cutoff energy and k-point number were set to be 300.0 eV and 2 $\times $ 5 $\times $ 2, respectively. Oscillator strength and corresponding UV-vis spectra are calculated by TDDFT under PBE0 level implemented by the CP2K software package [38,39]. Cluster model calculation was carried out under PBE0 level with TDDFT implemented by the BDF software package [40–44].

## Supplementary Material

nwae180_Supplemental_File
