# Supplementary material for: Spin-configuration of emission states in zero-dimensional metal halides
Source: Natl Sci Rev. 2024 May 25;12(5):nwae180. doi: 10.1093/nsr/nwae180 (PMC11970236; doi:10.1093/nsr/nwae180)
Supplement: nwae180_Supplemental_File [file nwae180_supplemental_file.docx]

Supplemental Material

**Spin-Configuration of Emission States in Zero-dimensional Metal Halides**

Zhiyuan Kuang ^1^, Xinyu Huang ^1^,Xing Wang ^1^, Chengcheng Wang ^1^, Xinrui Wang ^1^, Wei Huang ^1,2^, Qiming Peng ^1*^ and Jianpu Wang ^1,3*^

^1^ *Key Laboratory of Flexible Electronics (KLOFE), Institute of Advanced Materials (IAM) & School of Flexible Electronics (Future Technologies), Nanjing Tech University (NanjingTech), 30 South Puzhu Road, Nanjing 211816, China.*

^2^ *Strait Laboratory of Flexible Electronics (SLoFE), Fuzhou 350117, China.*

^3^ *Changzhou University, 21 Middle Gehu Road, Changzhou 213164, China.*

**Correspondence to:*

*iamqmpeng@njtech.edu.cn (Qiming Peng)*

*iamjpwang@njtech.edu.cn (Jianpu Wang)*


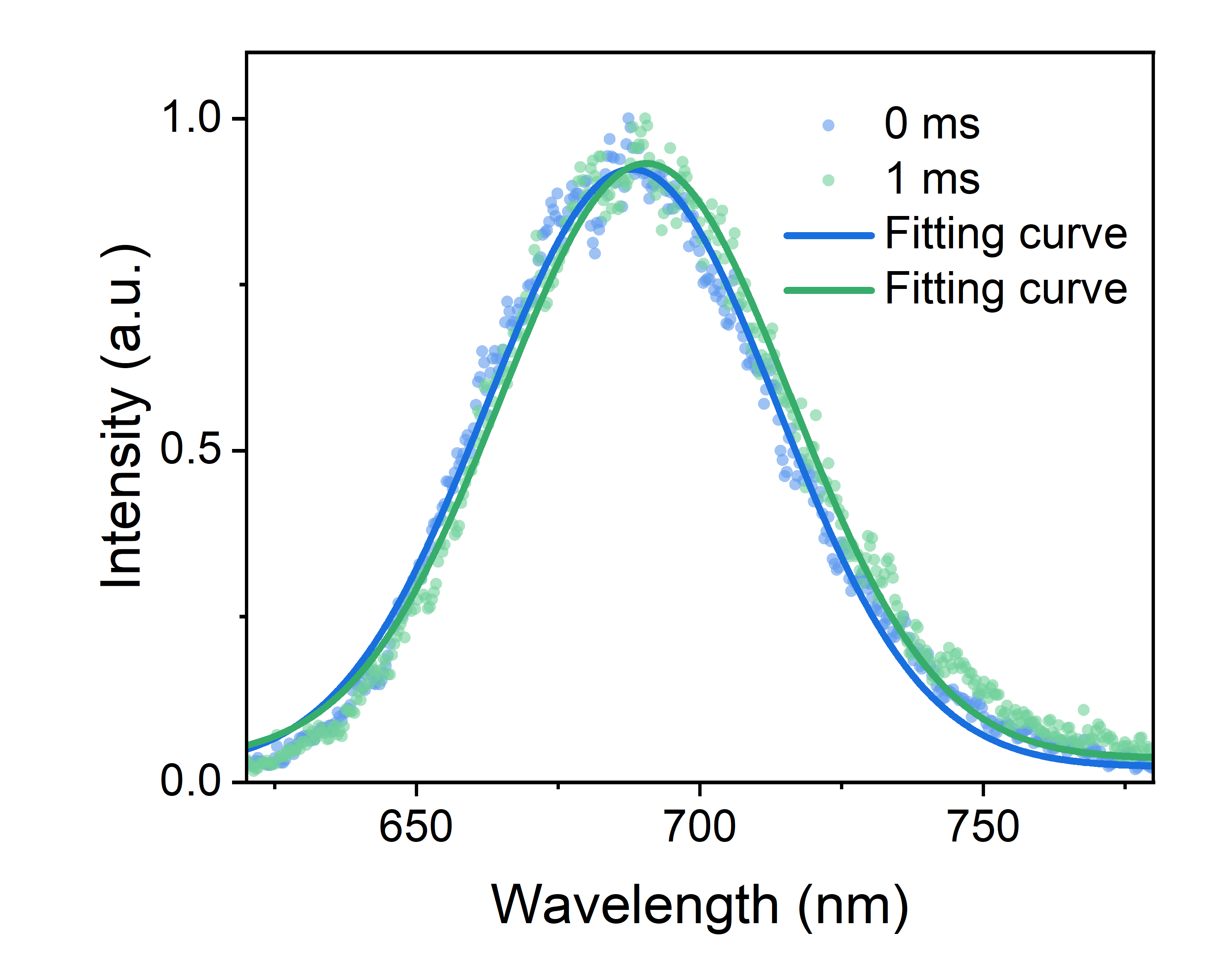


**FIG. S1.** PL spectra of Peak 1 at 0 and 1 ms at 0 T and 2 K.


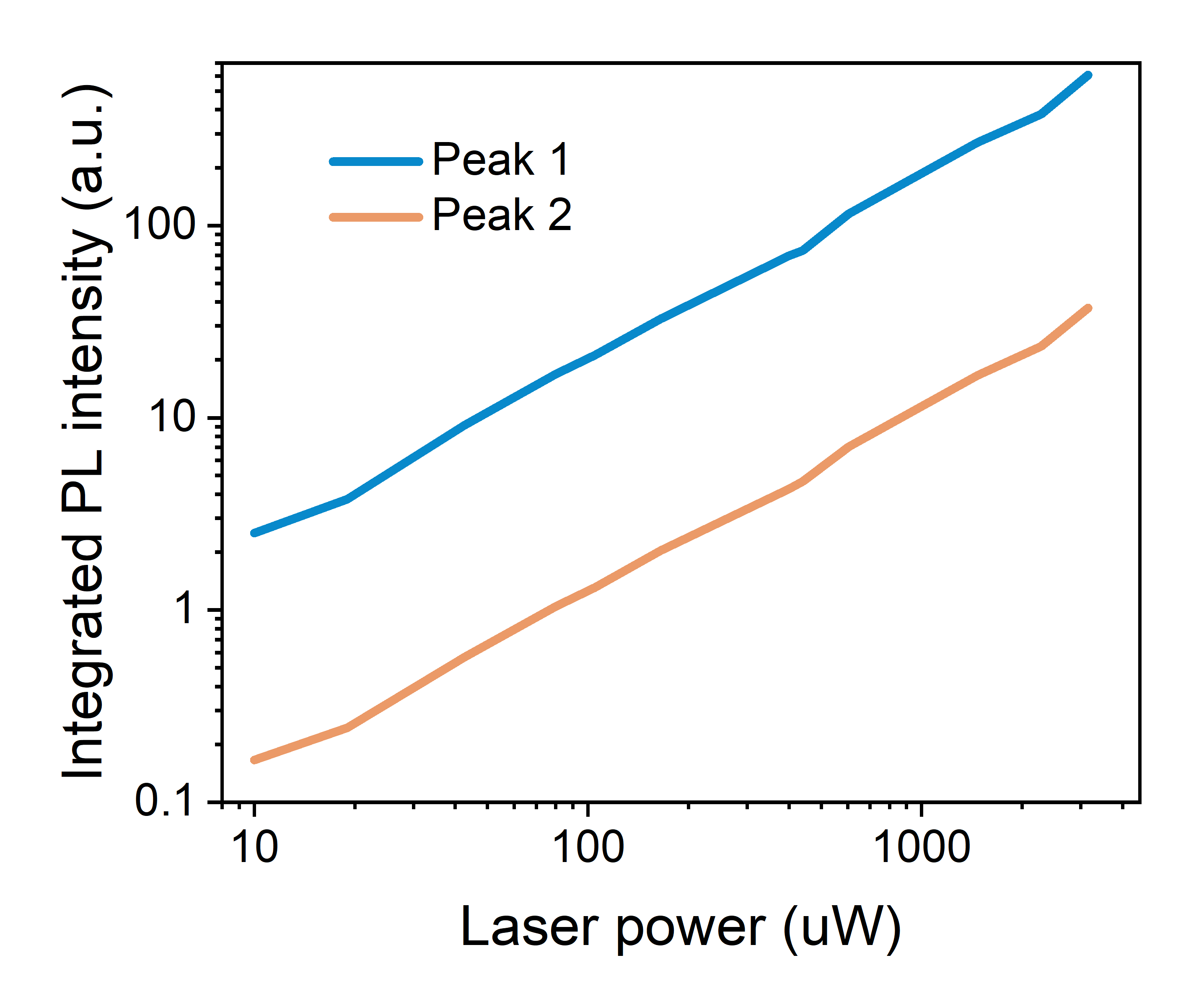


**FIG. S2.** Integrated PL intensities of Peak 1 and Peak 2 under different excitation intensities.

**
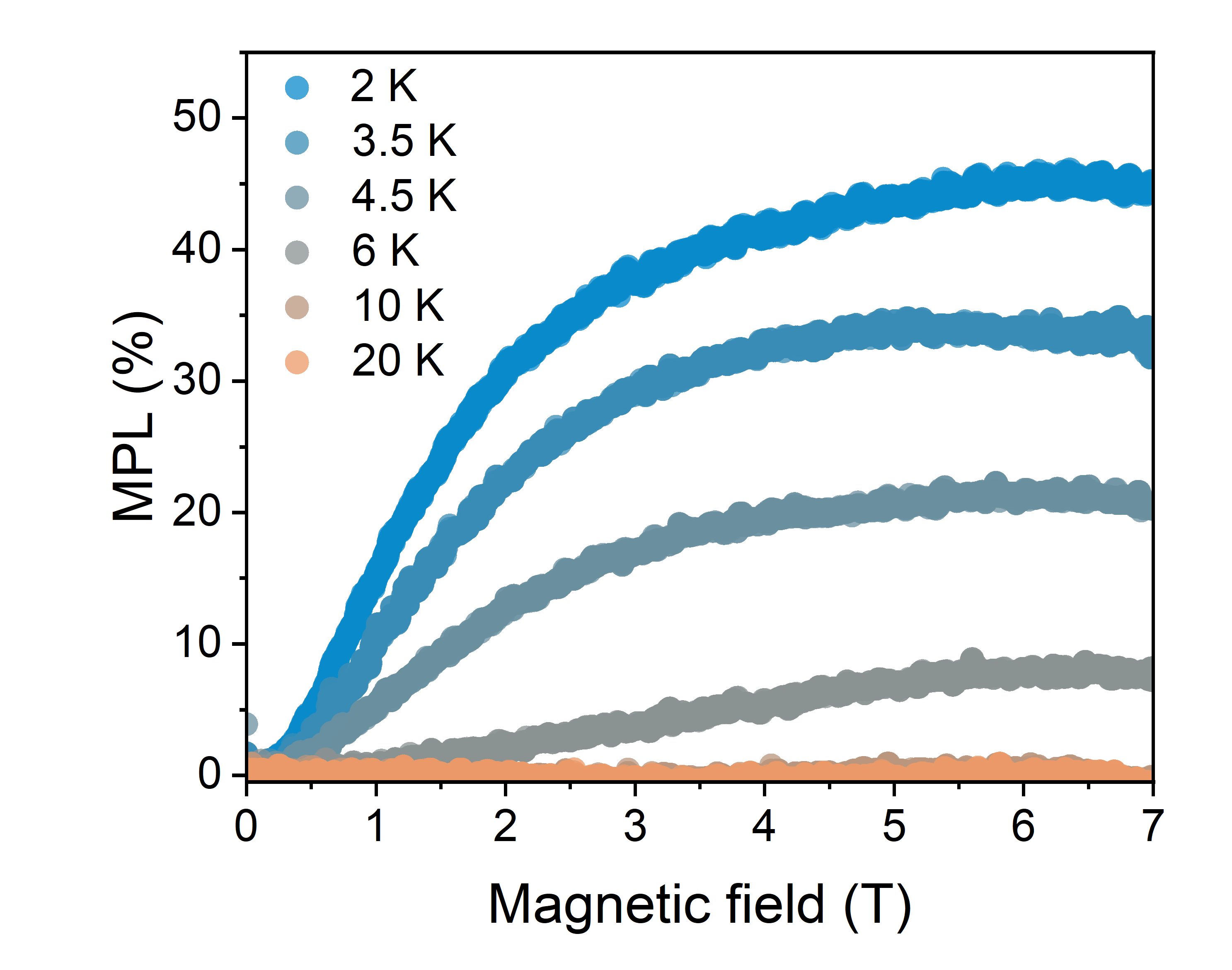
**

**FIG. S3.** MPL of Peak 1 at different temperatures vary from 2 to 20 K.


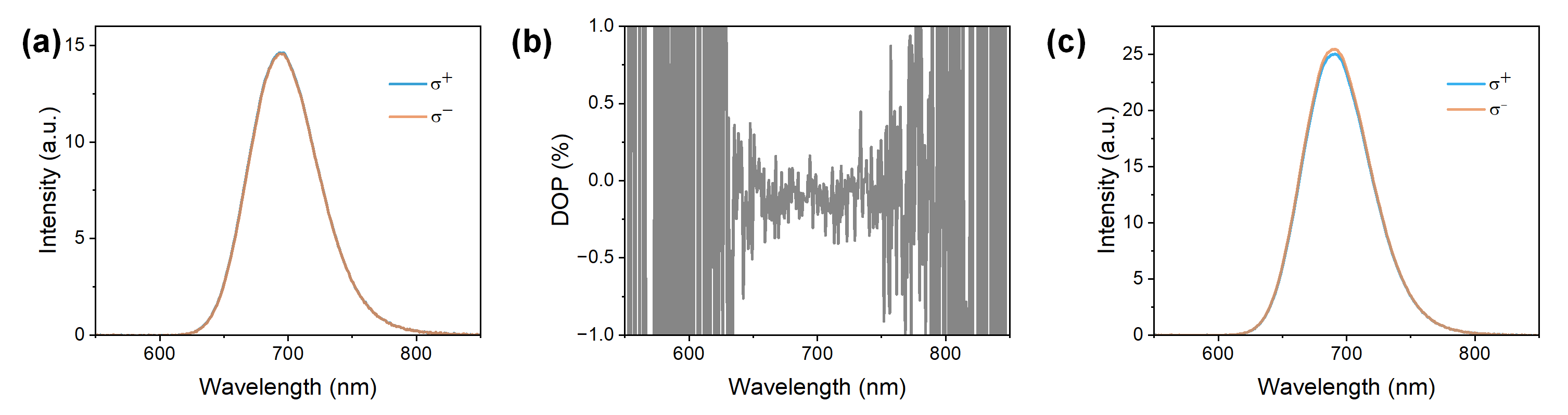


**FIG. S4.** (a) $\sigma^{+/-}$ PL spectra at 2 K 0 T. (b) DOP of (Bmpip)2SnBr4 at 2 K and 0 T. (c) $\sigma^{+/-}$ PL spectra at 2 K 7 T.


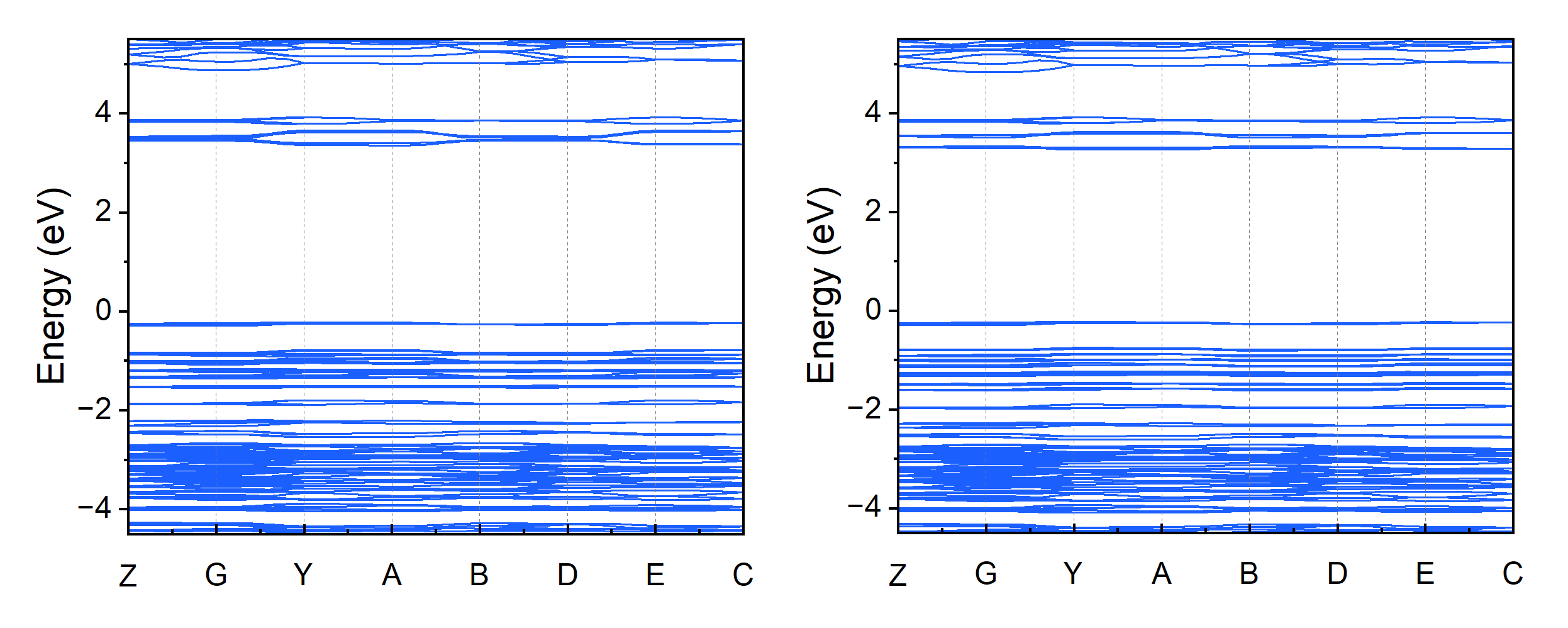


**FIG. S5.** Calculated band structure without (a) and with (b) the concerning of spin-orbital coupling (SOC).


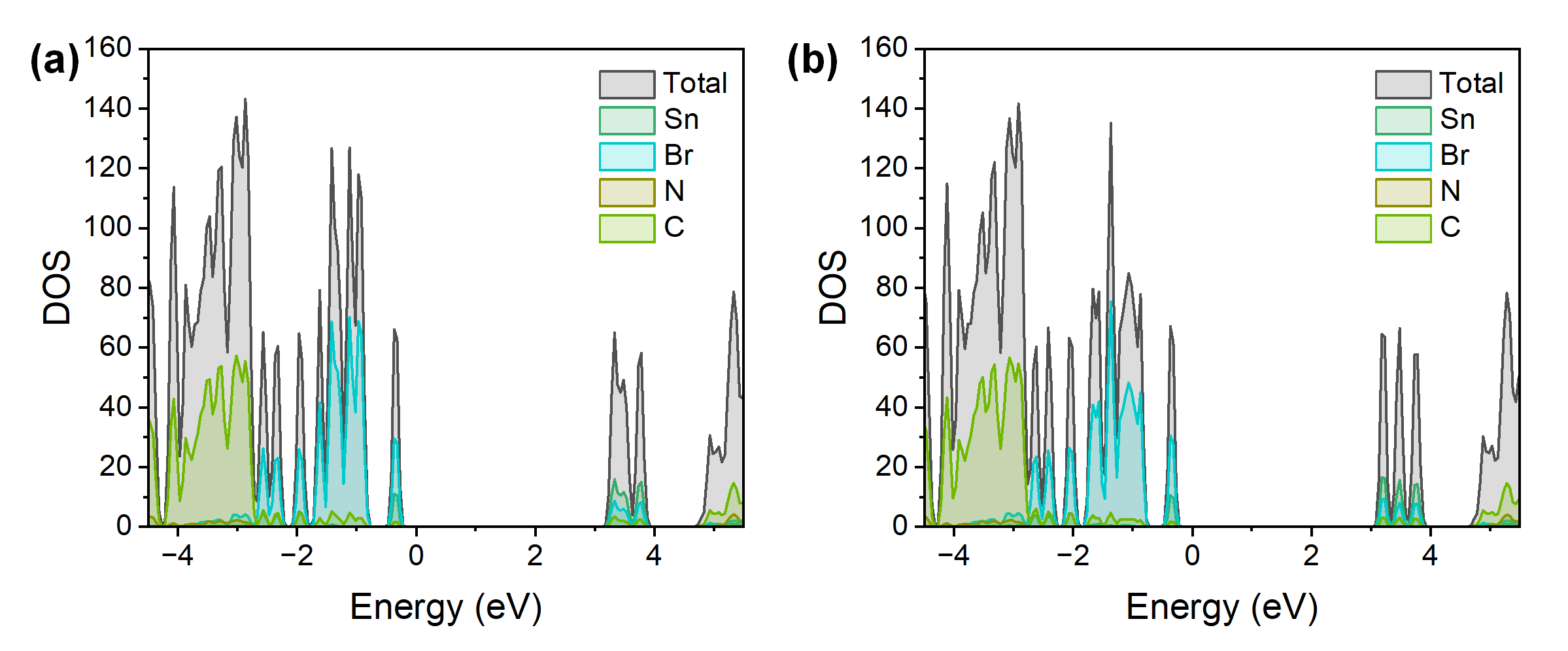


**FIG. S6.** Calculated density of states without (a) and with (b) the concerning of SOC.


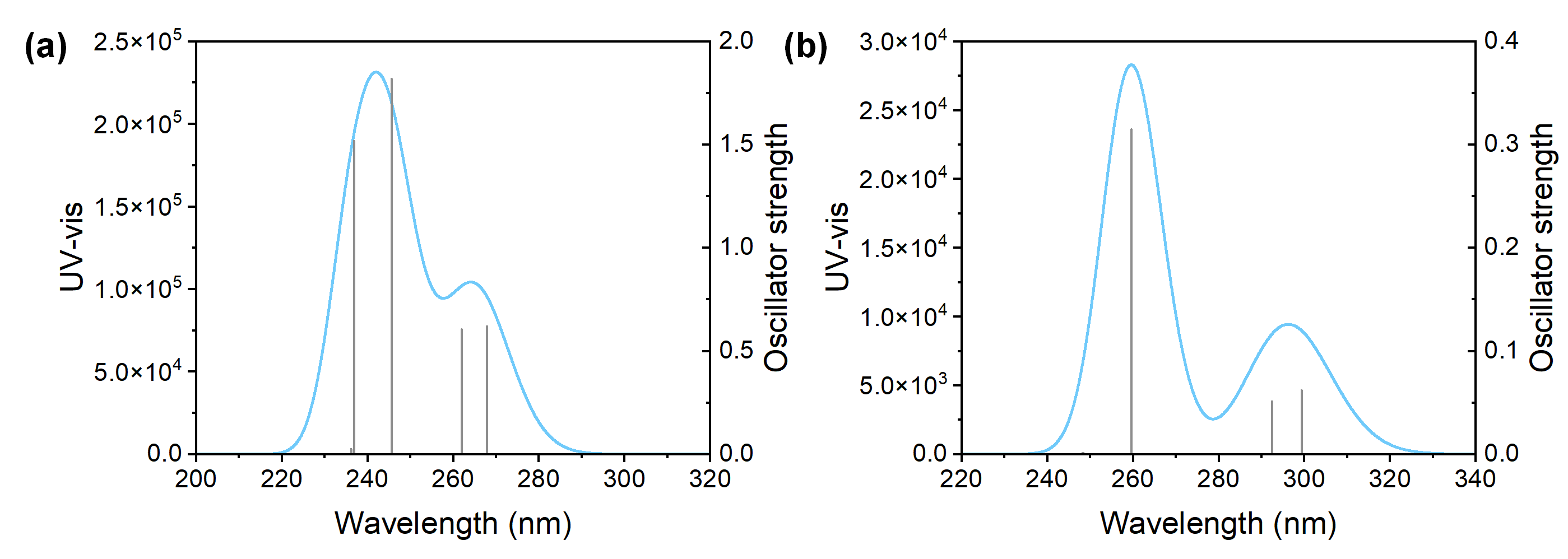


**FIG. S7.** (a) Calculated UV-vis spectrum and oscillator strength under TDDFT level implemented in CP2K package. (b) Calculated UV-vis spectrum and oscillator strength under TDDFT level with cluster model implemented in BDF software.


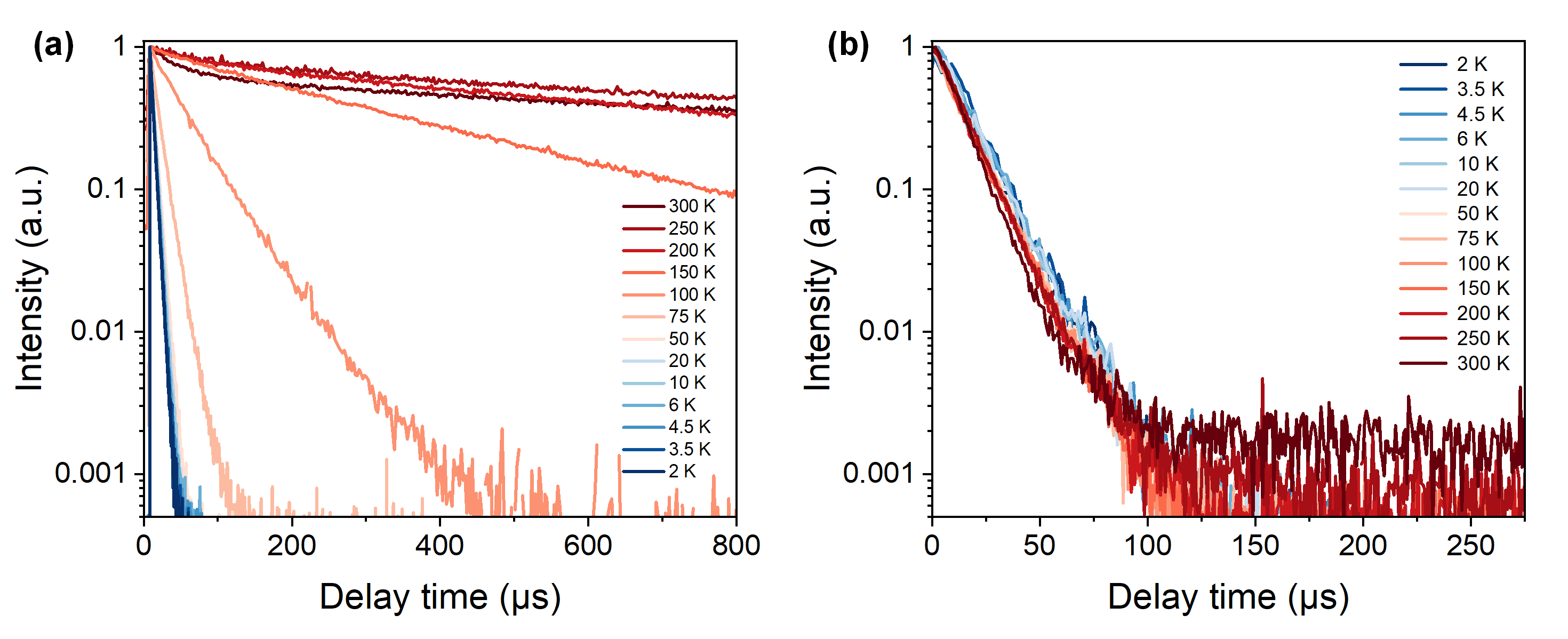


**FIG. S8.** TRPLs of Peak 1 (a) and Peak 2 (b) from 2 to 300 K.
